# The effect of a novel anticonvulsant chemical Q808 on gut microbiota and hippocampus neurotransmitters in pentylenetetrazole-induced seizures in rats

**DOI:** 10.1186/s12868-022-00690-3

**Published:** 2022-02-03

**Authors:** Xiang Li, Qing Wang, Di Wu, Dian-wen Zhang, Shu-chang Li, Si-wei Zhang, Xia Chen, Wei Li

**Affiliations:** 1 Department of Pharmacology, College of Basic Medical Sciences, Jilin University, Changchun, Jilin China; 2 Academy of Chinese Medical Sciences of Jilin Province, Changchun, Jilin China; 3 Jilin Cancer Hospital, Changchun, Jilin China

**Keywords:** Seizure, Gut microbiota, Neurotransmitter, Q808

## Abstract

**Background:**

The gut microbiota can modulate brain function and behavior and is increasingly recognized as an important factor in mediating the risk of epilepsy and the effects of seizure interventions. Drug therapy is one of the factors that influence the composition of the intestinal microbiota. Q808 is an innovative chemical with strong anticonvulsant activity and low neurotoxicity. However, studies evaluating the effect of Q808 on gut microbial communities are lacking. In this study, we aimed to evaluate the anticonvulsant activity of Q808 on a pentylenetetrazol (PTZ)—induced seizure model and analyze and compare the intestinal microbiota composition of non-PTZ vehicle control group, the PTZ-induced seizure model rats with and without Q808, through 16S rDNA sequencing. Neurotransmitter levels in the hippocampus were quantitatively estimated using HPLC–MS.

**Results:**

The results suggest that Q808 effectively alleviates seizures in chronic PTZ-kindled model rats. Additionally, based on the analyzed abundance of the gut microbiota, dysbacteriosis of model rats was found to be corrected after Q808 treatment at the phylum level. The unique bacterial taxa (e.g., *Lactobacillus*) that are associated with acetylcholine production, were significantly increased. Several short-chain fatty acids (SCFAs)-producing bacteria, including *Roseburia, Alloprevptella*, *Prevotellaceae_NK3B31_group*, *Prevotellaceae_UCG-001*, *and Prevotella_9*, were enriched. In the hippocampus, the contents of acetylcholine increased, whereas the levels of 3-methoxytyramine, glutamine, and 5-hydroxyindole acetic acid (5-HIAA) decreased after Q808 treatment.

**Conclusions:**

This study demonstrates that Q808 can be used to remodel the dysbiosis of the gut microbiome and influence neurotransmitter levels in the hippocampus of PTZ-induced seizure model rats. We hope that these novel findings prompt further research on the interaction between gut microbiota and seizures and the mechanism of Q808.

**Supplementary Information:**

The online version contains supplementary material available at 10.1186/s12868-022-00690-3.

## Background

Epilepsy is a severe neurological disease that affects more than 50 million people of all ages worldwide, and an estimated 2.4 million patients are diagnosed with epilepsy every year [1]. Epilepsy often requires lifelong medication and places an enormous burden on individuals and society [2].

Recent researchers have showed that dysbiosis is often found in conjunction with central nervous system (CNS) diseases, such as Parkinson’s disease, multiple sclerosis, and epilepsy [3]. At the same time, there is mounting evidence that gut microbiota could affect the occurrence and development of CNS disease. For example, depletion of the gut microbiome caused by antibiotic treatment or germ-free mice increases susceptibility to seizures and is associated with alterations in memory, sociability, and cognition [4]. These behavioral alterations can be restored by recolonizing the complete microbiota or specific microbes [5]. Transferring the gut microbiota of a person suffering from a CNS disease to animals through stool transplantation can facilitate the transfer of disease symptoms, such as depression [6]. Thus, utilizing the gut microbiota to maintain brain function and behavior is promising. Emerging studies have indicated that reconstruction of the gut microbiota can have a positive effect on epileptic seizures [7]. For example, some studies showed an almost 50% reduction in seizure frequency in patients with refractory epilepsy after probiotic treatment or ketogenic diet, which poses a significant effect on imbalanced gut microbiota [8, 9]. Other studies suggested a beneficial effect of healthy donor fecal microbiota transplantation (FMT) in disease symptoms and pathogenesis in epilepsy [10]. These animal studies and clinical cases have suggested a potential associations between gut microbiome and epileptic seizures.

Neurotransmitters play an important role in epilepsy. For example, an imbalance of excitatory and inhibitory neurotransmitters may lead to epileptic seizures [11]. Gut microbes regulate central neurotransmitter metabolism directly or indirectly through host biosynthetic pathways and can influence CNS diseases [12]. In a recent study, the enrichment of *Akkermansia* and *Parabacteroides* were shown to affect hippocampal γ-aminobutyric acid (GABA)/glutamate ratios and restore protection from seizures [4]. Additionally, chronic treatment with *Lactobacillus rhamnosus JB1* changes GABA mRNA expression in the mouse brain [13], and acetate, the main metabolite produced by microbes, changes the contents of glutamine, glutamate, and GABA in the hypothalamus [14]. Overall, factors that can modify the construction of gut microbiota may have the potential to regulate neurotransmitter levels in the brain and influence CNS disease.

Pharmacological treatment, diet, and other external factors, such as infection and psychological and physical stressors, can disturb the gut microbiota, thus can be associated with the risk of epilepsy [1]. A study of 1197 medications showed that nearly a quarter of non-antibiotic drugs across all classes inhibit the growth of at least one of the 40 known bacterial strains [15]. To date, few studies have investigated the relationship between anticonvulsant drugs and the gut microbiota. Lamotrigine, a traditional anticonvulsant drug, exhibits antibacterial activity against gram-positive bacteria, such as *Bacillus subtilis* and *Streptococcus faecalis* [16]. Valproate significantly increases the levels of *Clostridium *sensu stricto* 1* and *Ruminiclostridium 5* and decreases the relative abundance of *S24-7 uncultbact* [17]. A few cohort studies have reported the mild effects of antiseizure treatments, such as carbamazepine, on gut microbes [18]. These studies highlight the need to consider drug-induced changes in the gut microbiota.

**Fig. 1:**
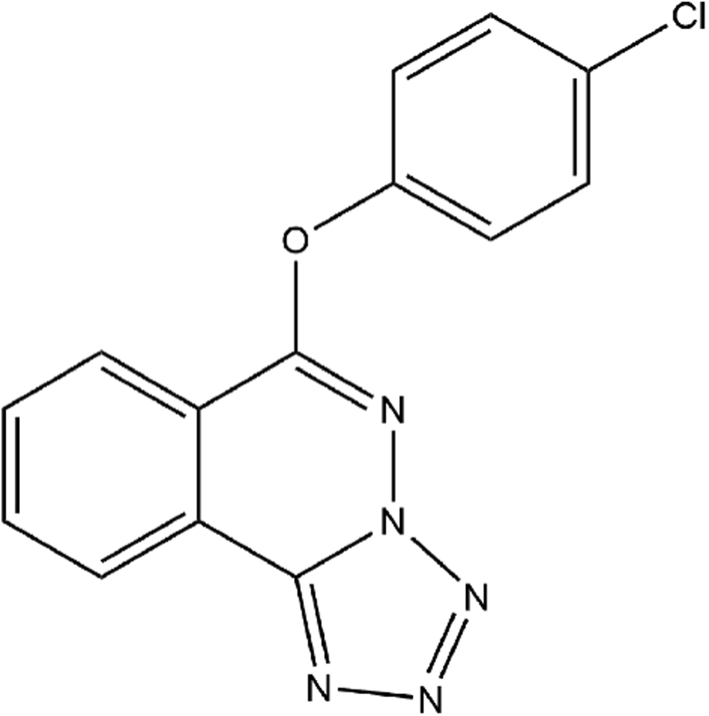
The chemical structure of Q808

Q808 [6-(4-chlorophenoxy)-tetrazolo(5,1-a)phthalazine] is an innovative compound that has exhibited potent anticonvulsant activity in a maximal electroshock mouse seizure model and has been shown to provide resistance against seizures induced by PTZ, ISO, THIO, and 3-MP [19]. The chemical structure of Q808 is shown in Fig. 1. Our previous study showed that Q808 can increase GABA levels in the hippocampus [20]. However, the level of other neurotransmitters and changing in the gut microbiota induced by Q808 remain unknown. The current study aimed to sequence the 16S rDNA in the fecal contents of a PTZ-induced seizure model in rats to understand the effects of Q808. Moreover, seizure levels and neurotransmitter contents in the hippocampi of PTZ-induced model rats with and without Q808 were determined.

## Methods

### Experimental animals and drugs

A total of 50 male Wistar rats (body weight 180–200 g; age 7–8 weeks; SPF level) were purchased from Beijing Vital River Laboratory Animal Technology Co., Ltd., China. Two rats were housed per polycarbonate cage in an SPF laboratory animal room under controlled conditions (temperature: 25 ± 2 °C; humidity: 50–60%). All animals were fed a standard diet and water ad libitum. After a 1-week adaptation period, the mice were randomly allocated to different research groups. Q808 was gifted to us from the Academy of Chinese Medical Sciences of Jilin Province. The other drugs and reagents were purchased from Sigma-Aldrich Chemical Company (St. Louis, MO, USA).

### PTZ kindling and seizure scoring

PTZ was freshly diluted in 0.9% NaCl, and a sub-convulsive dose (35 mg/kg, intraperitoneal) was injected every other day for 28 days. One week after the last injection of PTZ, it was again administered to the rats for another five consecutive days. Rat behaviors were evaluated blindly by observation for 30 min after the PTZ injection and scored according to Racine’s scale evaluation as follows: Phase 0: no evidence of convulsive activity; Phase 1: mouth and facial movements; Phase 2: head nodding; Phase 3: facial twitching, forelimb clonus; Phase 4: generalized clonic convulsions; Phase 5: falling and loss of righting reflex. The animals that were scored as phase 3, 4, or 5 for at least three consecutive evaluations were considered to be completely kindled [21].

### Drug administration and experimental design

The rats were divided into two groups. The ten rats in the vehicle control group received saline. The remaining forty rats were injected intraperitoneally with 35 mg/kg PTZ to establish an epileptic seizure model. After the model was successfully established (success rate, 60%), the rats were randomly allocated into either the PTZ + vehicle group or the PTZ + Q808 group (12 rats per group). The PTZ + Q808 group received Q808 over the following 28 days via oral gavage daily. The vehicle control group and PTZ + vehicle group received the solvents of Q808 at the same time and same route as the PTZ + Q808 group. Q808 drug solutions were freshly prepared with Tween-80 and 0.5% CMC-Na, and 30 mg/kg was administered to the rats via oral gavage daily. The body weights of the experimental animals were measured every other day.

### Fecal and hippocampal sample collection

After the behavioral tests, fresh feces were collected from each animal. A sterile filter paper was placed on the test bench. Rat was caught gently and feces were snapped frozen in liquid nitrogen before storing at − 80 °C. The animals were then humanely euthanized by CO_2_ inhalation. The animals were decapitated, and the heads were transferred to dry ice. The hippocampi were immediately dissected and homogenized in nine volumes of cold saline to prepare 10% cerebral homogenates. All fecal and hippocampal samples were stored at − 80 °C for further analysis.

### DNA isolation and quality measurement

Total genomic DNA was extracted from the samples (wet weight 120 mg) using a DNA Extraction Kit (Tiangen Company, Beijing, China) following the manufacturer’s instructions. Quality and quantity control of DNA was detected using a Nanodrop and agarose gel.

### PCR and 16S rDNA gene sequencing

Extracted DNA was diluted to a concentration of 1 ng/μL and used as a template for PCR amplification of the bacterial 16S rDNA gene with barcoded primers and Takara Ex Taq. The primers 343F (5′-3′ TACGGRAGGCAGCAG) and 798R (5′-3′ AGGGTATCTAATCCT) were used to amplify the V3-V4 variable region of the 16S rDNA genes. The PCR products of sterile water were used as negative controls for 16S rDNA sequencing. PCR was performed using Bio-Rad. Cycling was performed using the following parameters: 5 min at 94 °C, followed by 26 cycles of denaturation (94 °C for 30 s), annealing (56 °C for 30 s), and extension (72 °C for 20 s), and a final extension at 72 °C for 5 min. The samples were identified by electrophoresis on a 1% agarose gel. The amplicons were sequenced using an Illumina MiSeq System (Illumina, California, USA).

### 16S rDNA microbial community analysis

Raw sequencing data were in FASTQ format. Paired-end reads were then obtained using Trimmomatic software to detect and remove ambiguous bases, and were merged using FLASH software. Sequence denoising was performed using QIIME software (version 1.8.0) with ambiguous reads, and homologous sequences, putative chimeric sequences, and sequences with < 200bps were removed. The sequences based on distance had a clustering structure to generate operational taxonomic units (OTUs) using Vsearch software according to 97% similarity. The representative reads of each OTU were selected using the QIIME package. All representative reads were annotated and blasted against the Silva database Version 123 using the RDP classifier (70% confidence threshold).

### Estimation of neurotransmitter

Levels of the main neurotransmitters, including GABA, glutamine, glutamate, 5-HIAA, normetanephrine, dopamine, histamine, norepinephrine, 3-methoxytyramine, acetylcholine, DOPA, epinephrine, 5-Hydroxy-l-tryptophan, tyramine, and serotonin in the hippocampus were estimated using the LC–MS method with Agilent 1290 Infinity LC (Agilent, USA) and 5500 QTRAP (AB SCIEX, USA). A BEH C18 column (particle size 1.7 μm; 2.1 mm × 100 mm i.d.; Waters; Milford, USA) was used at 45 °C. Samples were injected and chromatographic separation was achieved with a mobile phase that consisted of two solvent (A and B). Solvent A contained formic acid (0.1%) and ammonium formate (25 mM), and solvent B consisted of acetonitrile containing 0.1% formic acid. The gradient elution of solvent B was performed using the following program: 0–18 min, 90–40%; 18–18.1 min, 40%–90%; 18.1–23 min, 90%. The flow rate was set at 300 μL/min, and the injection volume was approximately 2 μL. Mass spectrometry signals were collected using the positive ion (ESI +) scanning mode. The main parameters were as follows: source temperature, 450 °C; ion source gas 1 (Gas1), 60; ion source gas 2 (Gas2), 60; curtain gas, 30; and ion spray voltage floating, 5000 V. The multiple reaction monitoring mode was used.

### Statistical analysis

Prism version 5.00 (GraphPad Software, Inc., USA) was used for analysis. The continuous variables such as body weight was presented as the mean ± SEM values and compared between groups using one-way ANOVA followed by Tukey’s test. The neurotransmitter contents were presented as the mean ± SEM values and analyzed using the Kruskal–Wallis and Dunn’s non-parametric test. Seizure score (a discrete variable) was analyzed with Mann–Whitney *U* test. Indexes of α-diversity (Chao 1 index) were analyzed by the Kruskal–Wallis test. Differences in relative abundances of OTUs were calculated using Tukey's honest significance test by R package. β-diversity was calculated by the pMANOVA analysis based on Bray Curtis distances. Statistical significance was set at *p* < 0.05.

## Results

### Scores of PTZ kindling seizure model

**Fig. 2:**
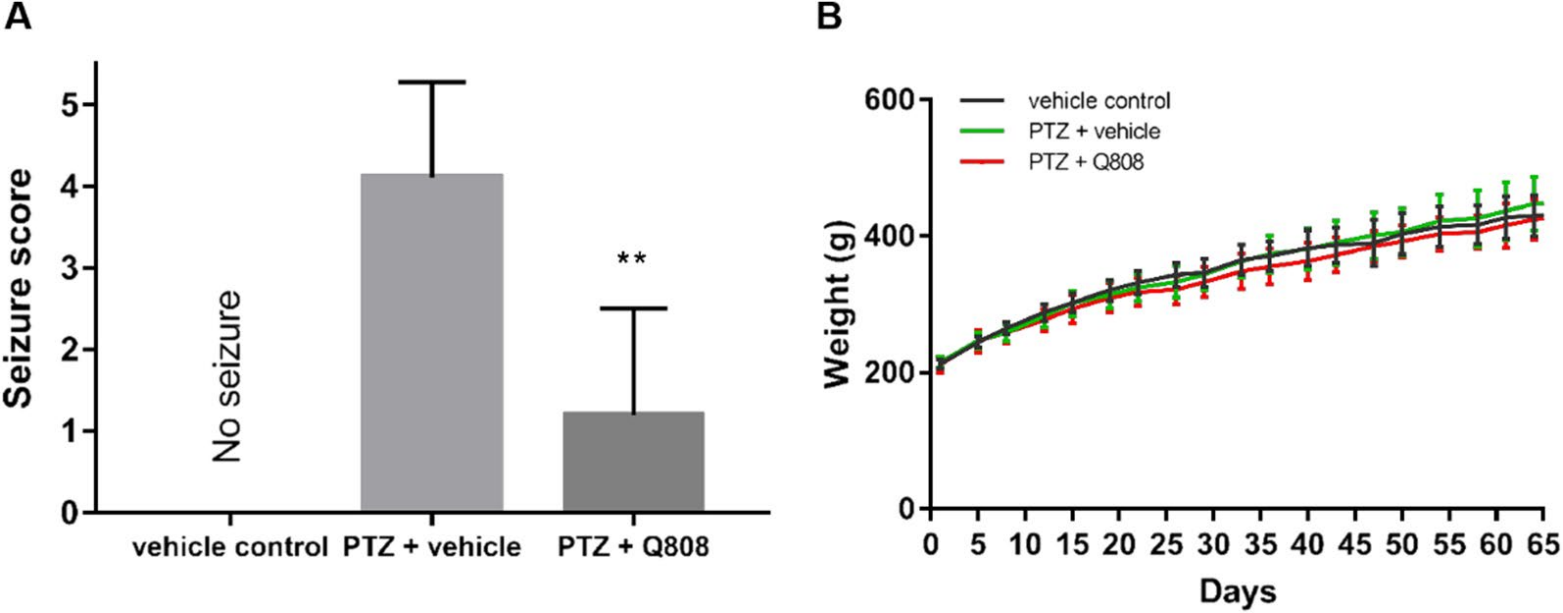
Effects of Q808 on PTZ kindling-induced **A** seizure stage and **B** body weight. Q808 effectively alleviates seizures in PTZ-kindling model rats (*p* = 0.003). The body weight of rats in each group have no difference. These data were shown as mean ± SEM and seizure score was analyzed with Mann–Whitney *U* test. **p < *0.01*

PTZ-induced seizure model rats were successfully established by repetitive administration of a sub-convulsive dose of PTZ for 28 days. The kindled rats were then administered Q808. PTZ was then injected again for 5 days, seizure intensity was scored according to the Racine’s scale evaluation, and mean seizure phases were calculated (Fig. 2A). The data demonstrated that Q808 significantly reduced the mean seizure score compared to the model group, indicating that Q808 had an excellent anticonvulsant effect on the PTZ-induced seizure model. No significant changes in the rat’s body weights were observed during the PTZ injection and drug administration process (Fig. 2B).

### Within-sample microbial diversity in seizure models with and without Q808

**Fig. 3:**
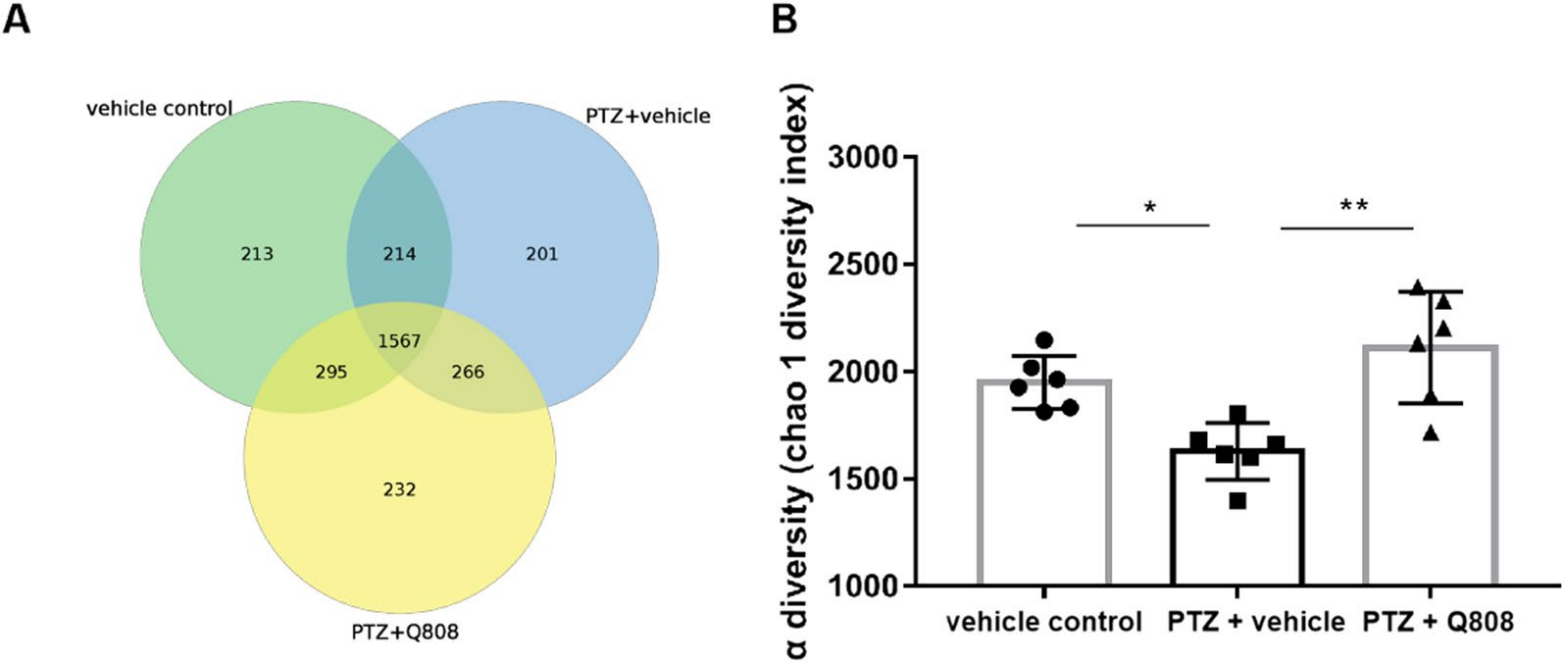
**A** Venn diagram and **B** α-diversity analysis for control, model, and Q808 groups (n = 6). Venn diagram illustrates the average unique and common OTU numbers in three groups. The α-diversity (Chao 1 index) showed that the diversity of the gut microbial community was significantly decreased in model group compared with control (*p* = 0.021) and significantly increased in Q808 group compared with model group (*p* = 0.001). Differences in relative abundances of OTUs were calculated using Tukey’s honest significance test by R package. Indexes of α-diversity (Chao 1 index) were analyzed by the Kruskal–Wallis test. *p < *0.05*, **p < *0.01*

An average length of 411.81 reads per samples was obtained after filtering. Then all remaining reads were clustered into 2988 OTUs with 97% sequence similarity. The Venn diagram shows 1567 OTUs in all groups, with 213, 201, and 232 OTUs unique to the control, model, and Q808 groups, respectively (Fig. 3A). Most rarefaction curves, as shown in Additional file 1: Fig. S1, tended to reach the saturation plateau, indicating that the sequencing depth was sufficient to cover the entire bacterial diversity. Specifically, microbiota profiles from the model group exhibited lower α-diversity than those from the control group when measured using the Chao index for evenness, and α-diversity was significantly elevated after treatment with Q808 (Fig. 3B).

### Alterations in gut microbiota composition in the PTZ-induced epileptic model before and after Q808 treatment based on the 16S rDNA data

**Fig. 4:**
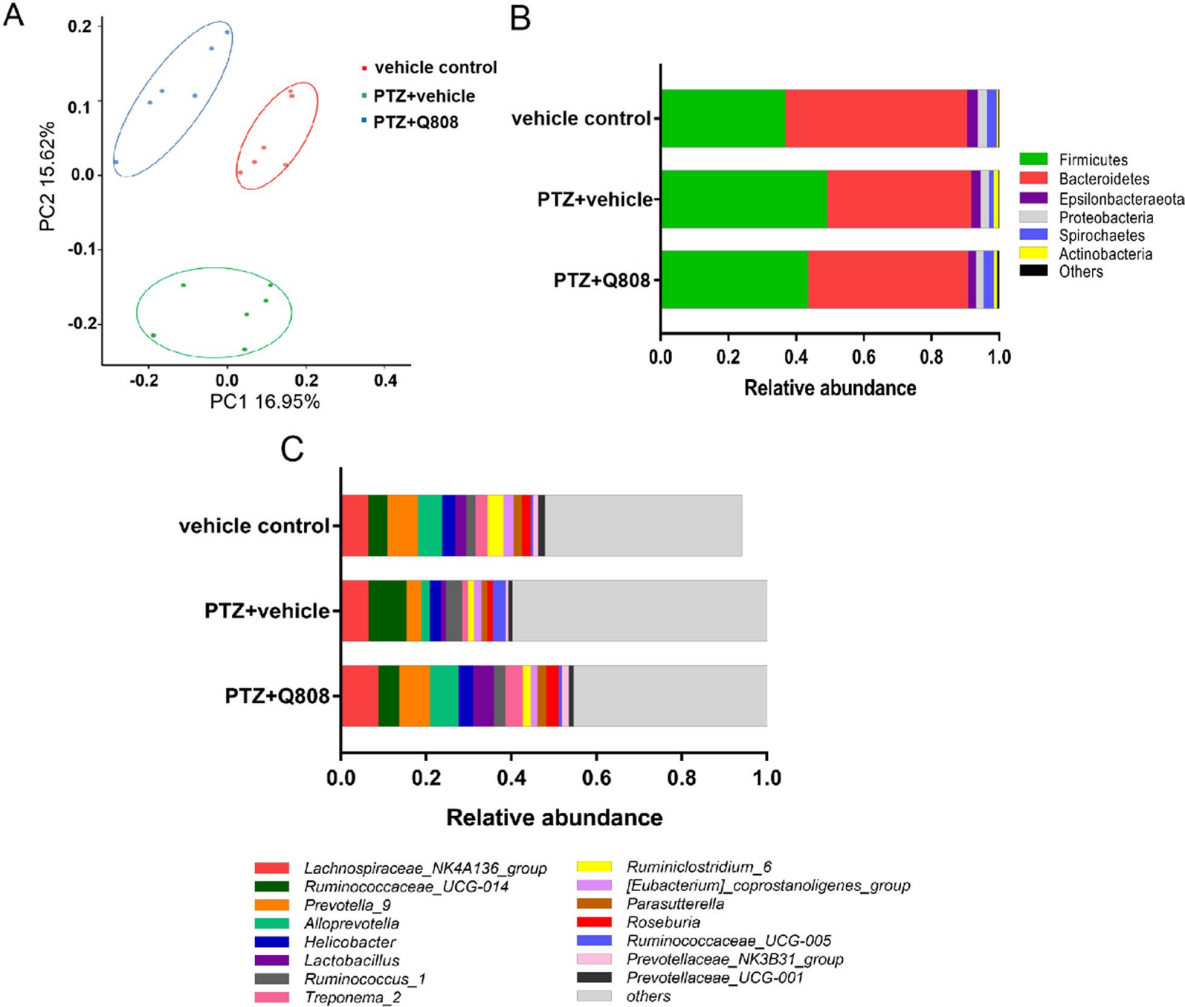
Alterations in the gut microbiota of PTZ-induced seizure model rats depending on Q808 treatment. **A** Two-dimensional principal coordinate analysis (PCoA) of the control, model, and Q808 groups. Each sample is marked by a dot. Samples that are more similar to one another are closer together. **B** Distribution of gut microbiota in the control, model, and Q808 groups at the phylum level. **C** Distribution of gut microbiota in the control, model, and Q808 groups at the genus level (n = 6). β-diversity was calculated by the pMANOVA analysis based on Bray Curtis distances

16S rDNA sequencing revealed distinct fecal microbiota alterations depending on the presence of Q808 treatment. Unweighted UniFrac analysis, which focuses on the diversity of gut microbiota (β-diversity), was used to evaluate the differences in species complexity among the groups. As shown in Fig. 4A, the three-dimensional plots of unweighted UniFrac analysis showed an obvious difference in the gut microbial community composition among the three groups. At the phylum level, reduced relative abundance of *Bacteroides, Epsilonbacteraeota,* and *Proteobacteria* and elevated abundance of *Firmicutes* and *Actinobacteria* were observed in the epileptic model group compared with the control group. However, dysbiosis of the gut microbiota in the epileptic model was reconstructed after treatment with Q808 (Fig. 4B). In other words, the levels of *Bacteroides*, *Epsilonbacteraeota,* and *Proteobacteria* increased, whereas those of *Firmicutes* and *Actinobacteria* decreased after the administration of Q808 for 28 days. Compared with the control group, the bacterial community profiles at the genus level showed that *Ruminococcaceae_UCG-014* and *Parasutterella* were highly enriched in the seizure model, whereas *Prevotella_9*, *Alloprevotella*, *Lactobacillus*, and *Roseburia* were lower in the model group. After treatment with Q808, the relative abundance of *Prevotella_9*, *Alloprevotella*, *Lactobacillu*s, and *Roseburia* increased, whereas the levels of *Ruminococcaceae_UCG-014* and *Parasutterella* significantly decreased (Fig. 4C, Additional file 1: Table S1).

### Neurotransmitters in the hippocampus

The level of 15 neurotransmitters were identified by LC–MS analysis. The extracted ion chromatograms of the neurotransmitter standards are shown in Additional file 1: Fig. S2. The metabolites were separated by chromatography, and each chromatographic peak was sharp and symmetrical, and could thus be used for mass spectrometry.

**Fig. 5:**
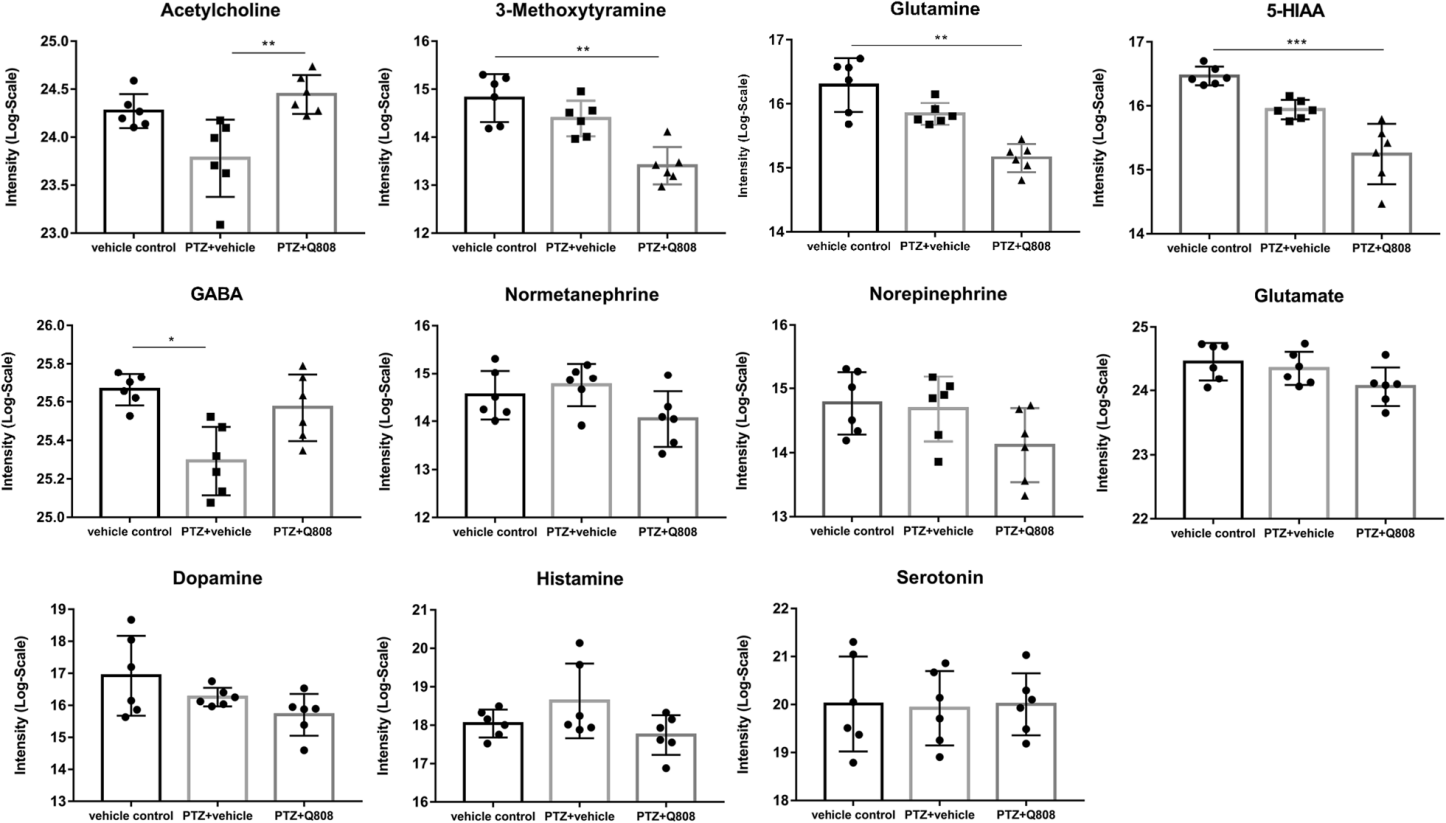
Normalized intensity of neurotransmitters in the hippocampi of the rats in three groups (n = 6)*.* In the seizure model group, the content of GABA was drastically decreased compared with that in control group (*p* = 0.011). After Q808 treatment, the content of acetylcholine increased (*p* = 0.002 compared with the PTZ group), whereas the levels of 3-methoxytyramine, glutamine, and 5-HIAA decreased (*p* = 0.003, *p* = 0.001, and *p* = 0.0004, separately, compared with the control group). No significant differences were observed in the levels of normetanephrine, norepinephrine, glutamate, dopamine, histamine, and serotonin among the three groups. Data were means ± SEM and analyzed using the Kruskal–Wallis and Dunn’s non-parametric test. *p < 0.05, **p < 0.01, ***p < *0.001*

As shown in Fig. 5, the content of GABA decreased significantly in the PTZ group compared with that in the vehicle control group. Acetylcholine, 3-methoxytyramine, glutamine, and 5-HIAA levels drastically changed after Q808 administration. 3-methoxytyramine, glutamine, and 5-HIAA levels decreased compared with those in the vehicle control group, whereas acetylcholine levels increased compared with that in the PTZ group. No significant differences were observed in the levels of normetanephrine, norepinephrine, glutamate, dopamine, histamine, and serotonin among the three groups. The contents of DOPA, epinephrine, tyramine, and 5-hydroxy-l-tryptophan were not detected.

## Discussion

The gut microbiota can modulate brain function and behaviors through the “microbiota-gut-brain” axis and may thus influence the onset of CNS diseases. Different interventions, such as pharmacological treatments, can exert considerable effects on the gut microbiota. In this paper, we reported alterations in the gut microbiota of PTZ-induced model rats before and after treatment with the novel anticonvulsant drug, Q808. The chemical kindling of seizures triggered by PTZ has been used to reflect the pathogenesis of human epilepsy and is considered an appropriate model for drug-resistant epilepsy [21]. The effect of Q808 on other models of epilepsy, such as kainic acid model, needs to be verified in the future. 16S rDNA gene sequencing, a deep sequencing technology, was used to analyze gut microbial communities. Our rat model of PTZ-kindled seizures showed a decrease in the relative abundance of *Bacteroidetes* and *Proteobacteria* and an increase in the abundance of *Firmicutes* and *Actinobacteria*, consistent with clinical reports on drug resistant epilepsy [22]. These four phyla are the main classes of microbiota, and an imbalance between their compositions is associated with epileptogenesis [1]. Supplementation with probiotics is frequently used in clinical practice [23]. Therefore, normalization of the intestinal microbiota is considered a novel treatment strategy for epileptic seizures. At the genus level, the abundance of different microbes changed in the model group. In a future study, we plan to determine whether changes in the abundance of specific microbes in the model group can increase susceptibility to seizures and whether these changes are biomarkers of epilepsy. In addition, the effect of discontinuation Q808 on seizures should be investigated in a future study.

Increasing evidence showed gut–brain interactions in neurological diseases [24], but only few studies have deeply focused on the relationship between gut microbiota and epilepsy. Q808 could be an effective scientific tool to help determine the role of gut microbiota in epilepsy. Q808 is an innovative anticonvulsant chemical with an international patent and is currently approved for clinical trials. After the administration of Q808, PTZ-induced epileptic seizures were effectively controlled, and the composition of the gut microbiota were nearly normal at the phylum level. In other words, Q808 showed anticonvulsant activity and restored the balance of the gut microbiota. The relative abundance of some probiotics, such as *Prevotella_9*, *Lactobacillus*, and *Roseburia* increased after treatment with Q808. Whether Q808 exerts an antiepileptic effect on gut microbiota and through the “microbiota-gut-brain” axis should be verified using the FMT method.

Recent studies have reported that the gut microbiota participates in the synthesis and metabolism of a wide range of neurotransmitters, including GABA and acetylcholine [25], and neurotransmitters play an important role in maintaining normal brain activity. In this study, we showed that the level of acetylcholine increases after treatment with Q808, and acetylcholine-producing bacteria, including *Lactobacillus* [12]*,* were enriched. In a recent study, probiotics such as *Lactobacillus rhamnosus* *GG (LGG)* were found to positively affect intestinal permeability [26]. However, whether the use of Q808 can restore intestinal permeability needs to be further explored. Asano’s group have reported that the norepinephrine levels of the cecal contents were lower in the GF mice than in the SPF mice, suggesting that gut microbes are a likely source for gut luminal norepinephrine [27]. In this study, Q808 decreased the norepinephrine levels in the hippocampus, and low levels of norepinephrine is associated with depression [28]. 5-HIAA is a metabolite of serotonin and is also associated with depression [29], and levels decreased after Q808 gavage in rats. These findings suggest that the use of Q808 may increase susceptibility to depression. Therefore, the combination of Q808 and serotonin-norepinephrine reuptake inhibitors may be a reasonable option for clinical use. In addition to the above neurotransmitters, 3-methoxytyramine (3-MT) and glutamine levels were altered after Q808 treatment. 3-MT is a metabolite of dopamine and a novel neuromodulator involved in movement control [30], and glutamine is the precursor of glutamate [31]. The gut microbiome of patients with schizophrenia modulates glutamine levels and schizophrenia-relevant behaviors in mice [32]. However, the specific gut microbes that produce these neurotransmitters in epilepsy is unknown. The associations between gut microbial structure and hippocampal neurotransmitter levels should be clarified by FMT or specific microbe supplementation in future studies. Gut microbes may not be the only influence on the response to neurotransmitter changes in the brain, although they are likely involved [12]. However, the relationship between gut microbiota and neurotransmitters after Q808 treatment remains unknown and needs to be further explored.

Metabolites contained within the transplant material, rather than the microbiome itself, can play a direct or indirect role in host behavior. SCFAs are the main metabolites produced by intestinal bacteria and are known to influence neuropsychiatric disorders. Animal studies have shown that SCFAs can cross the blood–brain barrier and alter the levels of neurotransmitters, including glutamate, glutamine, and GABA, and enzymes involved in the synthesis of neurotransmitters, and induce the transcription of neurotransmitters [33]. Interestingly, our results showed that the abundance of SCFA-producing bacteria, such as *Roseburia, Alloprevptella*, *Prevotella_9*, *Prevotellaceae_NK3B31_group*, and *Prevotellaceae_UCG-001* increased in the Q808 group. SCFAs exert an effect on appetite suppression through the vagus nerve, mediated by fatty acid receptor 3 [34]. Vagus nerve stimulation is an effective method to treat epilepsy. Thus, whether SCFAs provide protection against epilepsy should be explored. Q808 may be a good medium for elucidating the role of SCFAs in epilepsy.

## Conclusions

In this study, we provided seminal evidence that Q808 can reconstruct the composition of gut microbiota and increase probiotic levels in the gut. The levels of neurotransmitters in the hippocampus changed after Q808 treatment. Moreover, we found that microbial changes after treatment with Q808 were closely related to neurotransmitter production. Our findings provide a novel way to explore the mechanism of Q808 and provide novel insights into the relationship among microbiota, epileptic seizure, and anticonvulsant drugs.

## Supplementary Information

**Additional file 1: Fig. S1.** The Shannon and Simpson indices. The majority of the rarefaction curves tended to approach the saturation plateau, suggesting that the sequencing depth of the gut microbiome was sufficient for each group. **Fig. S2.** Extracted ion chromatograms of the 15 neurotransmitter standards. The metabolites were separated by chromatography, and each chromatographic peak was sharp and symmetrical. **Table S1. **16S rDNA sequencing results of rats in vehicle control, PTZ + vehicle, and PTZ + Q808 group.

## Data Availability

The datasets used and/or analyzed during the current study are available from the corresponding author on reasonable request.

## Abbreviations

PTZ: Pentylenetetrazol
SCFAs: Short-chain fatty acids
CNS: Central nervous system
GABA: γ-Aminobutyric acid
5-HIAA: 5-Hydroxyindole acetic acid
