# Supplementary material for: The effect of a novel anticonvulsant chemical Q808 on gut microbiota and hippocampus neurotransmitters in pentylenetetrazole-induced seizures in rats
Source: BMC Neurosci. 2022 Feb 3;23:7. doi: 10.1186/s12868-022-00690-3 (PMC8812211; doi:10.1186/s12868-022-00690-3)
Supplement: Supplementary file 1 — Additional file 1: Fig. S1. The Shannon and Simpson indices. The majority of the rarefaction curves tended to approach the saturation plateau, suggesting that the sequencing depth of the gut microbiome was sufficient for each group. Fig. S2. Extracted ion chromatograms of the 15 neurotransmitter standards. The metabolites were separated by chromatography, and each chromatographic peak was sharp and symmetrical. Table S1. 16S rDNA sequencing results of rats in vehicle control, PTZ + vehicle, and PTZ + Q808 group. [file 12868_2022_690_MOESM1_ESM.docx]

The effect of a novel anticonvulsant chemical Q808 on gut microbiota and hippocampus neurotransmitters in pentylenetetrazole-induced seizures in rats

Xiang Li^1^, Qing Wang^2^, Di Wu^2^, Dian-wen Zhang^2^, Shu-chang Li^3^, Si-Wei Zhang^1^, Xia Chen^1*^, Wei Li^2*^

^1^[Department of Pharmacology](https://vpn.jlu.edu.cn/http/77726476706e69737468656265737421e3e40f862f3972587b06c7af9758/s?sw=authorcompy%28Affiliations%2C1%2CDepartment+of+Pharmacology%29), [College of Basic Medical Sciences](https://vpn.jlu.edu.cn/http/77726476706e69737468656265737421e3e40f862f3972587b06c7af9758/s?sw=authorcompy%28College+of+Basic+Medical+Sciences%29), [Jilin University](https://vpn.jlu.edu.cn/http/77726476706e69737468656265737421e3e40f862f3972587b06c7af9758/s?sw=authorcompy%28Jilin+University%29), [Changchun, Jilin,](https://vpn.jlu.edu.cn/http/77726476706e69737468656265737421e3e40f862f3972587b06c7af9758/s?sw=authorcompy%28Changchun+130000%29) [China](https://vpn.jlu.edu.cn/http/77726476706e69737468656265737421e3e40f862f3972587b06c7af9758/s?sw=authorcompy%28China.%2C2%2CCardiac+Surgery+Department%29)

^2^ Academy of Chinese Medical Sciences of Jilin Province, Changchun, Jilin, China

^3^ Jilin cancer hospital, ChangChun, Jilin, China

*Corresponding author E-mail:

[chenxjluedu@163.com](mailto:chenxjluedu@163.com) (XC); [liwei611201@163.com](mailto:liwei611201@163.com) (WL)

**Additional file 1**

**
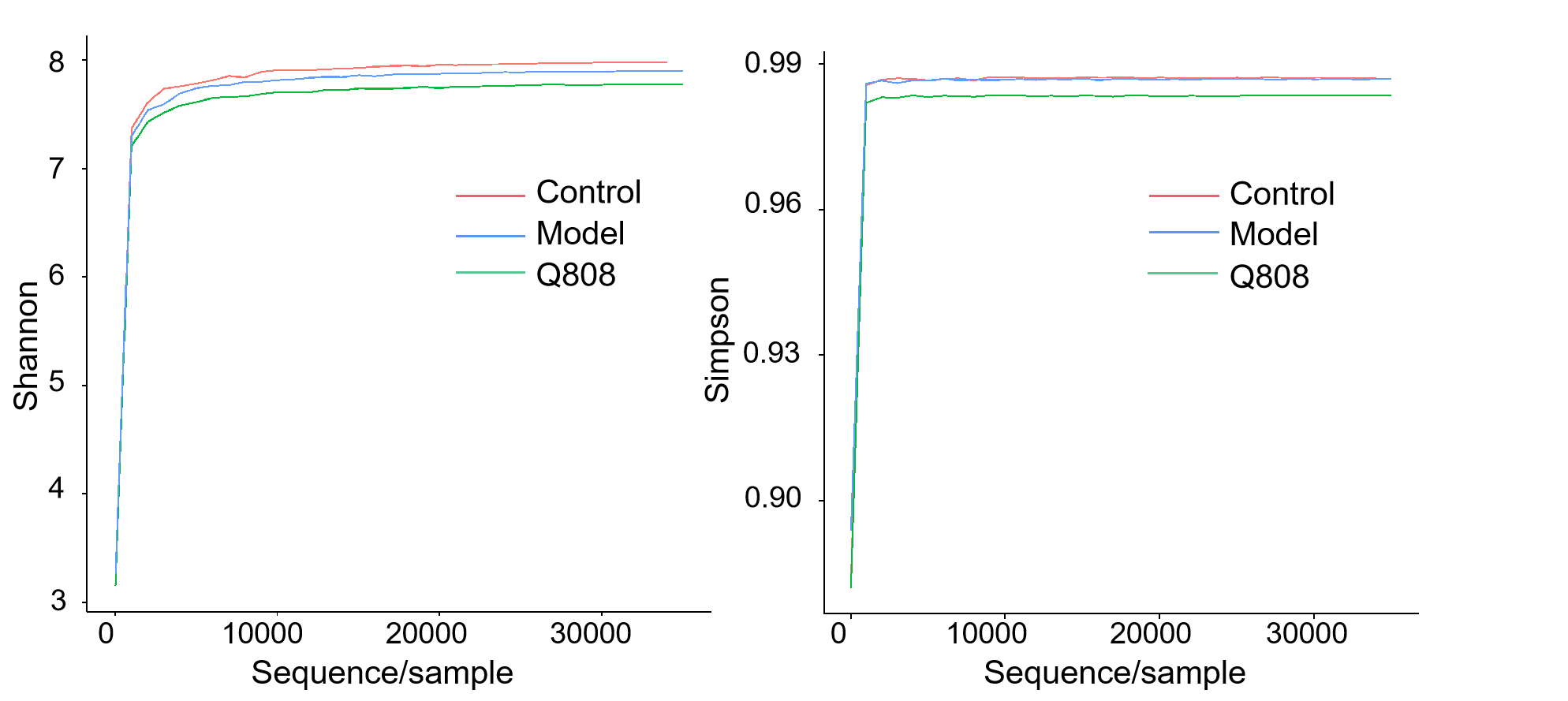
**

**Fig. S1.** **The Shannon and Simpson indices.** The majority of the rarefaction curves tended to approach the saturation plateau, suggesting that the sequencing depth of the gut microbiome was sufficient for each group.


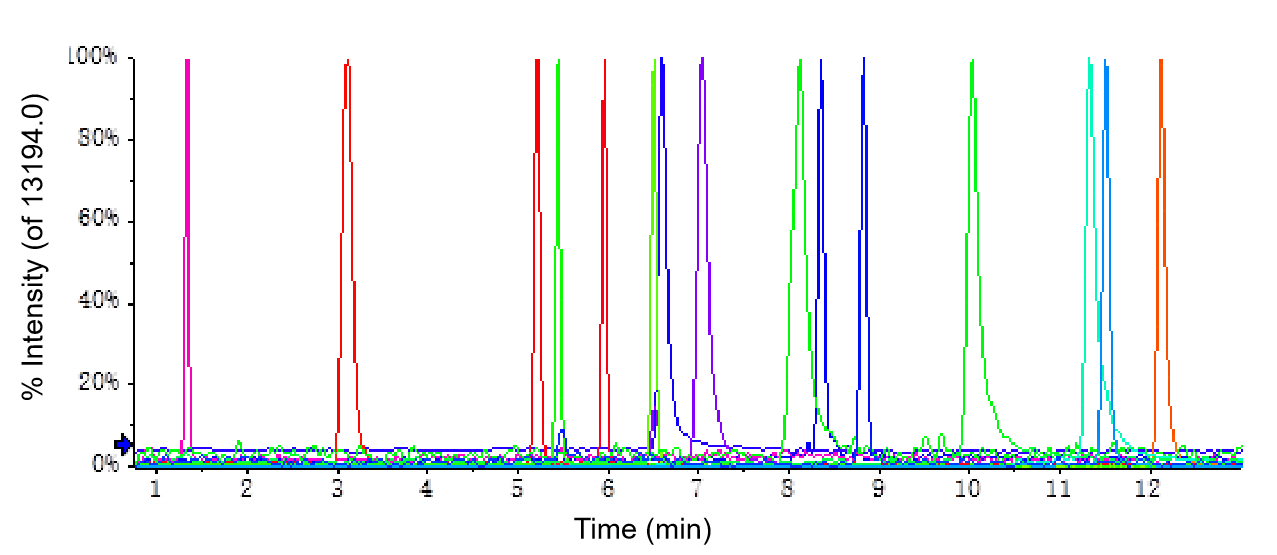


**Fig. S2.** **Extracted ion chromatograms of the 15 neurotransmitter standards.** The metabolites were separated by chromatography, and each chromatographic peak was sharp and symmetrical.

**Table S1. 16S rDNA sequencing results of rats in vehicle control, PTZ + vehicle, and PTZ + Q808 group.**

|  | **Vehicle control** | **PTZ + vehicle** | **PTZ + Q808** |
| --- | --- | --- | --- |
| **Lachnospiraceae_NK4A136_group** | 0.05944 | 0.05984 | 0.08383 |
| **Ruminococcaceae_UCG-014** | 0.04528 | 0.09001 | 0.04748 |
| **Prevotella_9** | 0.07151 | 0.03375 | 0.07274 |
| **Alloprevotella** | 0.05625 | 0.02032 | 0.06736 |
| **Helicobacter** | 0.03125 | 0.02601 | 0.03372 |
| **Lactobacillus** | 0.02615 | 0.01207 | 0.04919 |
| **Ruminococcus_1** | 0.02131 | 0.03845 | 0.02746 |
| **Treponema_2** | 0.02866 | 0.01289 | 0.04096 |
| **Ruminiclostridium_6** | 0.03608 | 0.01405 | 0.01836 |
| **[Eubacterium]_coprostanoligenes_group** | 0.02516 | 0.01787 | 0.01522 |
| **Parasutterella** | 0.01839 | 0.01391 | 0.02084 |
| **Roseburia** | 0.02060 | 0.01263 | 0.02873 |
| **Ruminococcaceae_UCG-005** | 0.00573 | 0.03067 | 0.00809 |
| **Prevotellaceae_NK3B31_group** | 0.01284 | 0.00611 | 0.01635 |
| **Prevotellaceae_UCG-001** | 0.01489 | 0.00873 | 0.01128 |
| **others** | 0.52646 | 0.60272 | 0.4634 |
